# Establishment of the early prediction models of low-birth-weight reveals influential genetic and environmental factors: a prospective cohort study

**DOI:** 10.1186/s12884-023-05919-5

**Published:** 2023-08-31

**Authors:** Satoshi Mizuno, Satoshi Nagaie, Gen Tamiya, Shinichi Kuriyama, Taku Obara, Mami Ishikuro, Hiroshi Tanaka, Kengo Kinoshita, Junichi Sugawara, Masayuki Yamamoto, Nobuo Yaegashi, Soichi Ogishima

**Affiliations:** 1 Department of Informatics for Genomic Medicine, Group of Integrated Database Systems, Tohoku Medical Megabank Organization, Tohoku University, 2-1, Seiryo-Machi, Aoba-Ku, Sendai, Miyagi 980-8575 Japan; 2 Department of Statistical Genetics and Genomics, Group of Disease Risk Prediction, Tohoku Medical Megabank Organization, Tohoku University, Miyagi, Japan; 3 Department of Molecular Epidemiology, Group of the Birth and Three-Generation Cohort Study, Tohoku Medical Megabank Organization, Tohoku University, Miyagi, Japan; 4 Medical Data Science Promotion, Tokyo Medical and Dental University, Tokyo, Japan; 5 Department of Statistical Genetics and Genomics, Group of Systems Bioinformatics, Tohoku Medical Megabank Organization, Tohoku University, Miyagi, Japan; 6 Department of Gynecology and Obstetrics, Tohoku University Graduate School of Medicine, Tohoku University, Miyagi, Japan; 7 Department of Feto-Maternal Medical Science, Group of Community Medical Supports, Tohoku Medical Megabank Organization, Tohoku University, Miyagi, Japan; 8 Suzuki Memorial Hospital 3-5-5, Satonomori, Iwanumashi, Miyagi 989-2481 Japan; 9 Department of Medical Biochemistry, Graduate School of Medicine, Tohoku University, Sendai, Japan; 10 Department of Integrative Genomics, Tohoku Medical Megabank Organization, Tohoku University, Miyagi, Japan

**Keywords:** Low birth weight, Machine learning, Artificial intelligence, Early prediction, Pregnancy, Environmental factors, Genetic factors

## Abstract

**Background:**

Low birth weight (LBW) is a leading cause of neonatal morbidity and mortality, and increases various disease risks across life stages. Prediction models of LBW have been developed before, but have limitations including small sample sizes, absence of genetic factors and no stratification of neonate into preterm and term birth groups. In this study, we challenged the development of early prediction models of LBW based on environmental and genetic factors in preterm and term birth groups, and clarified influential variables for LBW prediction.

**Methods:**

We selected 22,711 neonates, their 21,581 mothers and 8,593 fathers from the Tohoku Medical Megabank Project Birth and Three-Generation cohort study. To establish early prediction models of LBW for preterm birth and term birth groups, we trained AI-based models using genetic and environmental factors of lifestyles. We then clarified influential environmental and genetic factors for predicting LBW in the term and preterm groups.

**Results:**

We identified 2,327 (10.22%) LBW neonates consisting of 1,077 preterm births and 1,248 term births. Our early prediction models archived the area under curve 0.96 and 0.95 for term LBW and preterm LBW models, respectively. We revealed that environmental factors regarding eating habits and genetic features related to fetal growth were influential for predicting LBW in the term LBW model. On the other hand, we identified that genomic features related to toll-like receptor regulations and infection reactions are influential genetic factors for prediction in the preterm LBW model.

**Conclusions:**

We developed precise early prediction models of LBW based on lifestyle factors in the term birth group and genetic factors in the preterm birth group. Because of its accuracy and generalisability, our prediction model could contribute to risk assessment of LBW in the early stage of pregnancy and control LBW risk in the term birth group. Our prediction model could also contribute to precise prediction of LBW based on genetic factors in the preterm birth group. We then identified parental genetic and maternal environmental factors during pregnancy influencing LBW prediction, which are major targets for understanding the LBW to address serious burdens on newborns' health throughout life.

**Supplementary Information:**

The online version contains supplementary material available at 10.1186/s12884-023-05919-5.

## Background

Low birth weight (LBW) is defined as a birth weight of less than 2500 g [1] which affects approximately 6–20% of all neonates [1], and a leading cause of neonatal morbidity and mortality and various disease risks. The well-known risk factors for LBW are both environmental factors, including obstetric complications [2], maternal age, socioeconomic factors [2] and nutrition status [2], and genetic factors of both the mother [3] and fetus [4]. LBW is a high-impact disease with health effects across life stages because LBW is a risk factor for cognitive impairment [5] and physical development delay [6] in childhood and hypertension [7] and mental disorders [8] in adulthood. Because of these impacts of LBW, the development of early predictions and interventions for LBW is needed.

The development of early onset and treatment prediction of diseases are the most important targets of precision medicine for the early intervention and prevention of diseases [9, 10]. Of note for multifactorial diseases, the development of early prediction models is expected based on combining genetic and environmental factors [11] because of the involvement of the interplay of both environmental and genetic factors. In previous studies, AI models to predict LBW using maternal features [12] and models to predict small for gestational age (SGA) using fetal ultrasonography data [13] were developed; however, these studies have the following critical limitations: (1) small sample sizes of a few hundred subjects, (2) absence of genetic factors that are important risk factors, and (3) no stratification of neonates into preterm and term birth groups, which are critically different in the mechanism of the development of LBW.

The Birth and Three-Generation (BirThree) Cohort Study [14] of the Tohoku Medical Megabank (TMM) Project is a large-scale and unbiased multicenter prospective genome cohort study which are requirements for building generalized early prediction models. Birthree cohort study recruited more than 70,000 subjects, including more than 20,000 pregnant women and their children, partners and other family members from the regional population through 48 hospitals, including both advanced hospitals and urban/rural clinics in Miyagi Prefecture. The TMM BirThree Cohort Study collected and integrated both environmental and genetic factors including laboratory tests, genomic data, clinical records and lifestyle data [15] that are related to disease risk in many multifactorial diseases [16].

In this study, we challenged the development of early prediction models of LBW using large-scale environmental and genetic data of 20,000 subjects of the BirThree Cohort study. We stratified research subjects into preterm and term birth groups, and established the preterm LBW model and the term LBW model, respectively. We used health assessment data including self-report questionnaires and laboratory tests collected in the early stage of pregnancy as environmental data, and maternal and paternal SNP array data as genetic data. The developed artificial intelligence (AI) models were interpreted to identify influential features for prediction of LBW and differences between preterm and term birth groups.

## Methods

### Study design

We developed early prediction models of LBW using both environmental and genetic factors from the BirThree Cohort study. Because of the differences of the developing mechanism, we stratified subjects into preterm and term birth groups and developed early prediction models for each group. From the developed models, we identified influential features for prediction of LBW and differences between preterm and term birth groups.

### Data sources

The study population was selected from pregnancies, their partner, and neonates of the BirThree Cohort Study. BirThree cohort study recruited the regional population of the Miyagi Prefecture from 2013 to 2016 through 48 hospitals, including both advanced hospitals and urban/rural clinics. We used the following data collected in the BirThree cohort study to train early prediction models: 1) health assessment data, including maternal laboratory test data and questionnaire data in the early stages of pregnancy, 2) maternal and paternal SNP array data, and 3) fetal ultrasonography data. Among the data, we collected health assessment data and SNP array data based on uniform protocol in Tohoku University. As for fetal ultrasonography data, we collected data from daily medical practice as linkage data from 48 hospitals. The list of variables for health assessment data are provided in Supplemental table 1. SNP array data were genotyped by Japonica array v2 (JPAv2) [17] or Japonica array NEO (JPA NEO) [18]. The details of the processes of data collection and collected items in the BirThree cohort study are provided in a previous report [19]. In this study, we did not perform imputation or combine the two platforms. The list of all datasets used in this study is provided in Supplemental table 2.

### Study population

**Fig. 1:**
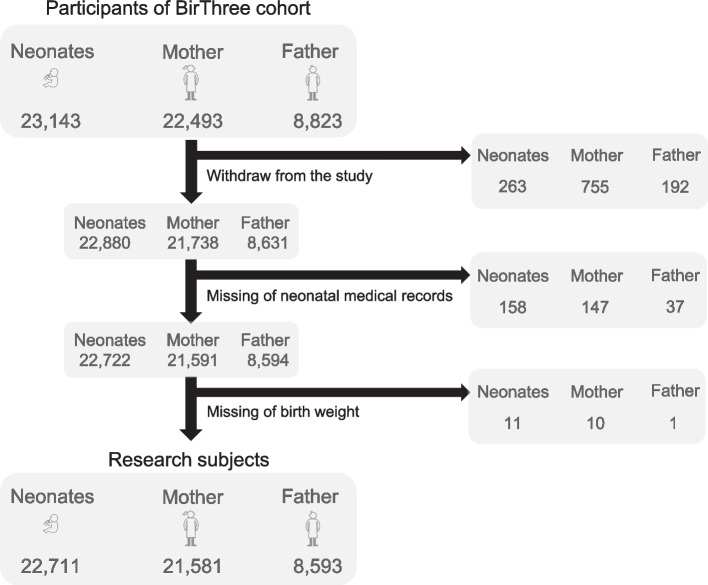
Selection of research subjects. From the 23,143 neonates and their parents, 22,711 neonates and their parents were selected using our selection criteria

We selected research subjects from 23,143 neonates included in the BirThree cohort study. We excluded neonates and their parents who withdrew from the study (*n* = 263), who did not have neonatal medical records (*n* = 158), and who had missing birth weight data (*n* = 11). Using these criteria, we included 22,711 neonates and their 21,581 mothers and 8,593 fathers in the dataset (Fig. 1).

### Scheme of our study

**Fig. 2:**
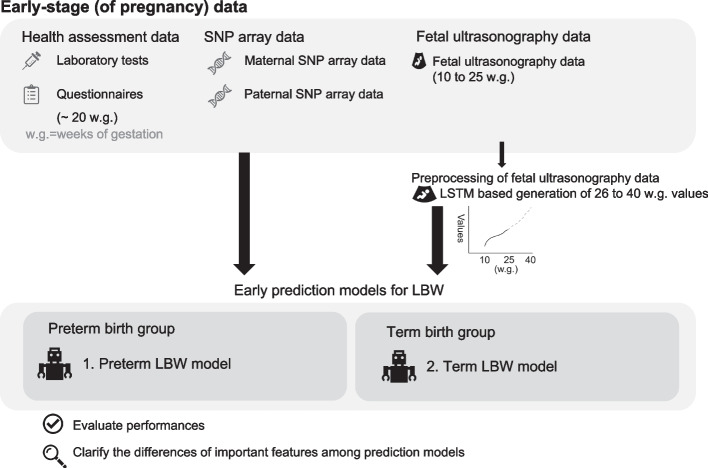
Scheme of our study. Health assessment data, SNP array data and fetal ultrasonography data were used as input of models. The values of fetal ultrasonography data in 26 to 40 weeks of gestation were predicted by AI to improve performance of models. We built the preterm LBW model and term LBW model which predict LBW in preterm birth and term birth group, respectively. The developed models were analyzed to identify influential factors of models and their differences between preterm and term LBW models

A scheme of our study is shown in Fig. 2. In this study, we developed two prediction models using data collected in the early stage of pregnancy as follows: the preterm LBW model predicts LBW in preterm neonates and their parents, and the term LBW model predicts LBW in term birth neonates and their parents. Stratification of preterm and term births was performed within 37 weeks of gestational age. For the early prediction model, we developed a total of 140 models consisting of a combination of 7 datasets, 2 feature selection methods, 1 sampling method, 5 learning models and 2 kinds of prediction models. Among the 140 models in the early prediction model, we interpret interpretable models to obtain feature importance for prediction. The obtained feature importance was used to clarify the differences in influential features for prediction among prediction models. The list of datasets for all models is provided in Supplemental Table 2. We also compared the prediction performance of the developed models with that of full-term data and controls to validate whether there is improvement or deterioration of the performance and whether there is any adverse effect including extreme overfitting (Supplemental Document). All analyses performed in this study are shown in Supplemental Fig. 1.

### Data preprocessing

In this study, we applied a series of preprocessing methods to health assessment data, including laboratory tests and questionnaires, time-series fetal ultrasonography data and SNP array data. In total, we built 7 datasets for the early prediction models.

The preprocessing of health assessment data was performed as follows: 1) building datasets of health assessment datasets, 2) imputation of missing values using multiple imputation by chained equations (MICE) [20], and 3) conversion of distribution by Box-Cox conversion [21] and/or scaling (see Supplemental Document).

As for fetal ultrasonography data, we performed preprocessing of 13 fetal ultrasonography items, including estimated fetal body weight (EFBW), as follows: 1) quality control (QC) of fetal ultrasonography data, 2) spline interpolation, 3) imputation of missing values by MICE, and 4) prediction of fetal ultrasonography data at the late stage of pregnancy (26–40 gestational weeks) using data in the early stage of pregnancy (10–25 gestational weeks) (see Supplemental Document).

As for SNP array data, we performed preprocessing of maternal, paternal and neonatal SNP array data genotyped by JPAv2 and JPA NEO as follows: 1) sample-based and probe-based QC, 2) dimension reduction of the SNP array data by pruning based on linkage disequilibrium and filtering of SNPs using *p*-values of genome-wide association analysis (see Supplemental Document).

### Development of early prediction models

We developed early prediction models for all health assessment data, fetal ultrasonography data and SNP array data as follows: 1) feature selection using the Hilbert–Schmidt independence criterion least absolute shrinkage and selection operator (HSIC-LASSO) [22] or recursive feature elimination (RFE), 2) under sampling of majority class until equal number of minority class for imbalanced learning, 3) training of the following five AI models including logistic regression (LR), random forest (RF), support vector machine (SVM), deep neural network (DNN) and extreme gradient boosting (XGBoost) [23], using either the health assessment data (laboratory tests or questionnaire data), fetal ultrasonography data, or SNP array data, and 4) interpretation of the interpretable LR, RF and XGBoost models to obtain feature importance for prediction.

To avoid overfitting, we performed tenfold internal–external cross validation by 10 times learning using 9/10 of the dataset as training/test data and remaining 1/10 of the dataset as validation data. We evaluate the performance of a developed model using the mean of the F1-score in 10 time learning and area under the curve (AUC). We then performed gene enrichment analysis on SNP array data to obtain summary weights and importance score of gene functions calculated from feature importance of interpretable models among developed models (see Supplemental Document).

## Results

### Proportion of datasets

We built 7 datasets consisting of health assessment data, SNP array and fetal ultrasonography data. The mean gestational ages at data collection of health assessment data were 20.64 (± 6.54) and 19.79 (± 7.93) for laboratory test and questionnaire data, respectively (Table 1).

**Table 1:** The number of features, subjects, and subjects with LBW in each dataset for the early prediction models

**Dataset**	**Number of features**	**Number of subjects**	**Number of subjects with LBW (n, %)**	**Mean gestational weeks of data collected (mean, SD)**
Laboratory test data collected in the early stage of pregnancy	27	22,035	1,975 (8.96)	20.64 (6.54)
Questionnaire data completed in the early stage of pregnancy	495	21,783	1,964 (9.02)	19.79 (7.93)
Maternal SNP array data (Japonica Array v2)	2,001	8,841	795 (8.99)	-
Maternal SNP array data (Japonica Array NEO)	2,155	9,845	952 (9.67)	-
Paternal SNP array data (Japonica Array v2)	2,001	3,005	283 (9.42)	-
Paternal SNP array data (Japonica Array NEO)	2,155	2,866	253 (8.83)	-
Fetal ultrasonography data	93	16,672	1,175 (7.05)	-

### Proportion of subjects

We identified 2,327 (10.22%) LBW neonates and their 2,140 mothers and 827 fathers. The number of preterm births was 1,397, including 1,077 (77.09%) LBW neonates. The number of term births was 21,042, including 1,248 (5.93%) LBW neonates. We identified 927 mothers and 339 fathers as parents of preterm LBW neonates and 1,213 mothers and 488 fathers as parents of term LBW neonates.

### The term and preterm LBW models showed high performance based on health assessment data and SNP array data, respectively

**Fig. 3:**
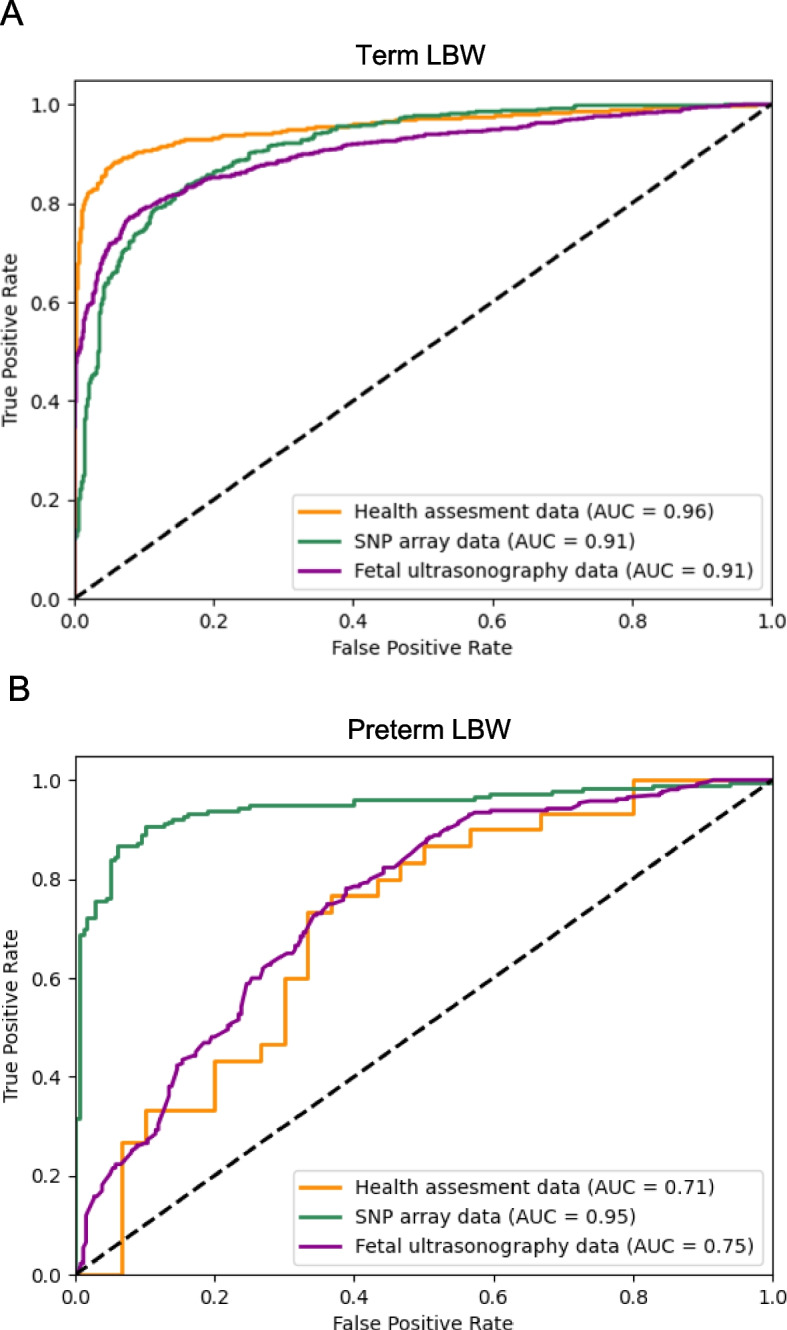
Prediction performance of the early prediction models of LBW. **A** In the term birth group, the model showed the highest performance with health assessment data (F1-score of 0.95) and modest performance with both SNP array data and fetal ultrasonography data (F1-score of 0.84 for both). **B** In the preterm birth group, the model based on the SNP array data (F1-scores of 0.90) showed the highest performance. The models based on other data, including health assessment data and fetal ultrasonography data, showed insufficient performance (F1-scores of 0.71 and 0.69, respectively)

We developed generalized early prediction models of term and preterm LBW using the BirThree cohort data consisting of an unbiased population. To develop prediction models, we conducted two feature selection methods, HSIC LASSO and RFE, for 7 datasets to build 14 variable lists for term and preterm birth groups, respectively (see Supplemental Table 3). The performance of the term LBW models showed high performance with F1-scores of 0.80–0.95 based on health assessment data and moderate performance based on parental SNP array and fetal ultrasonography data with F1-scores of 0.54–0.84 and 0.63–0.84, respectively. The AUC of the most high performance models among the term LBW models were 0.96, 0.91 and 0.91 for health assessment data (questionnaire data in the early stage of pregnancy), SNP array data and fetal ultrasonography data, respectively (Fig. 3A). For the term LBW models based on the health assessment data, the F1-scores using questionnaires (0.87–0.95) were higher than those of the models using laboratory tests (0.80–0.86) (Supplemental table 4). These results showed that maternal lifestyles are the most influential for predicting LBW in the term birth group.

Unlike the term LBW model, the preterm LBW model showed high performance with F1-scores of 0.58–0.90 based on maternal SNP array data and insufficient performance with F1-scores of 0.52–0.71 and 0.58–0.65 based on health assessment and fetal ultrasonography data, respectively. Note that we evaluated the performance of only models based on maternal SNP array data because we observed overfitting in the well performed model based on paternal SNP array data (Supplemental Table 5). The AUC of the most high performance models among the preterm LBW models were 0.71, 0.95 and 0.75 for health assessment data (laboratory test data in the early stage of pregnancy), SNP array data and fetal ultrasonography data, respectively (Fig. 3B). These results showed that only maternal SNP array data could be predictors of LBW in the preterm birth group.

### Interpretation of the high-performance term LBW models reveals that eating habits is the most influential health assessment category for prediction

**Fig. 4:**
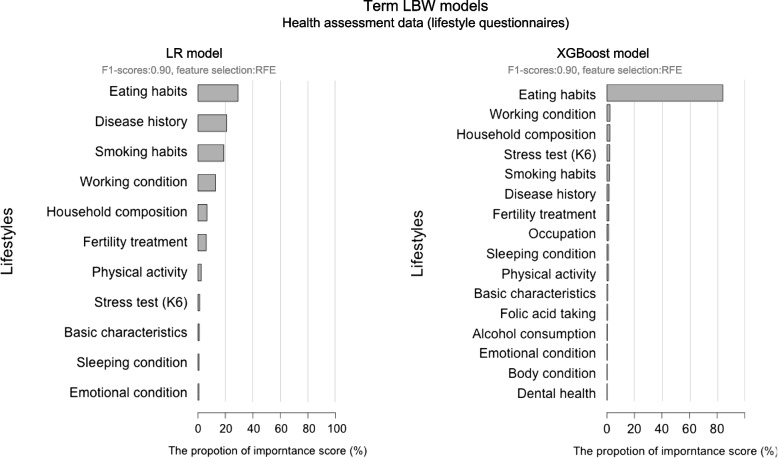
Proportions of importance scores. The proportions of importance scores of the models with the first and second highest prediction performances among interpretable models based on health assessment data in the term birth group. Eating habits showed the highest importance among comprehensive environmental factors in both models

The interpretation of the developed models was performed based on the first and second highest performance interpretable LR, RF and XGBoost models among the five AI models. For the term LBW models, we interpret the LR and XGBoost models which are the first and second highest performing models (F1-scores are 0.90 for both models). As a result, we identified the importance for the prediction for each variable (Supplemental Table 6) which were selected by feature selection from the all variables (Supplemental Table 1). The Supplemental Table 6 also provides the prioritized ranking based on the contributions to the model for the features in the first and second highest performing interpretable models. To clarify the characteristics of the health assessment variables, we classified the variables into categories as in Supplemental Table 6. We also summarized the importance of the categories of health assessment variables in Fig. 4. As shown in Fig. 4, the interpretation reveals that eating habits showed dominant high importance, which was 29.17–84.16%. Based on the dominant high importance, we identified eating habits as the most influential health assessment category. Except for eating habits, disease history (20.92%), smoking habits (18.69%), and working conditions (12.72%) had high importance in the LR model. For eating habits, we classified variables into sub-categories (see Supplemental Table 6). The proportion of the importance for sub-categories of features about eating habits were as follows: daily intake of foodstuffs (67.3–78.6%), frequencies of consuming foodstuffs (17.6–25.8%) and other features (3.8–6.9%). Based on the dominant high importance, we identified daily intake of foodstuffs as the most influential sub-category among eating habits. As shown in Supplemental Table 6, the high importance variables are different between the first and second highest performed interpretable models. In the highest performing LR model, disease history (e.g., endometriosis and polycystic-ovary syndrome), occupations (e.g., product sales, chef and medical technician), working conditions (e.g., number of times handling isotope during this pregnancy), and content of fertility treatment showed high importance. While in the second highest performing XGBoost model, daily intake of foodstuffs showed high importance, especially the intake of fish (e.g., sea bream and eel) and vegetables (e.g., papaya and broccoli).

### The interpretation of term LBW models based on SNP array data reveals that variants related to fetal growth are influential for prediction

The term LBW models showed the best performance based on the lifestyle data described above and moderate performance based on SNP array data. Based on the SNP array data, the two top interpretable models showed F1-scores of 0.76 and 0.74 for the models using maternal SNP array data and F1-scores of 0.68 and 0.69 for models using paternal SNP array data. The importance, gene types and nearest gene names for the variants based on the SNP array data in the early prediction models are shown in Supplemental Table 7. Gene enrichment analysis of variants except in intron regions, in no gene regions and in noncoding intron regions revealed that gene functions relating to insulin secretion and response to estrogen have a high importance in the term LBW models (Table 2). The GO terms from the first to the tenth importance score are shown in Table 2. All results of gene enrichment analysis for the term LBW model are shown in Supplemental Table 8. This result showed that variants related to fetal growth in the maternal or paternal genome were influential for predicting LBW in the term LBW group. The main cause of LBW in the term birth group is fetal growth restriction (FGR), and these variants should be involved in the development of FGR via the following mechanisms. The variants related to insulin secretion have an effect on the promotion of fetal growth through inheritance from parents to the fetus. The variants related to the response to estrogen are included in maternal SNP array data (Supplemental Tables 7 and 8) and have an effect on fetal growth through placental and ovarian maturation.

**Table 2:** Genes and their GO terms of the responsible variants for the term LBW prediction

**GO terms**	**Genes**	**Variants**	**The proportion of Importance score (%)**
**Positive regulation of insulin secretion**	PRKCE, GLUL, GHRL	rs117394235, rs17034641, rs75701130, AX-40988767, AX-30300885	**8.47**
Neurotransmitter uptake	SLC38A1, GLUL	AX-30300885, rs7971437	7.61
Positive regulation of positive chemotaxis	VEGFA, F7	rs3093253, rs3025022, rs3025039	6.93
**Positive regulation of epithelial cell proliferation**	VEGFA, LAMB1, GLUL	rs20556, AX-30300885, rs3025039, rs3025022	**5.87**
Protein complex assembly	TCEB2, HES1, PPID, IL2RB	rs229489, rs4036, rs8396, rs2368048	5.68
Negative regulation of protein ubiquitination	N4BP1, NXN, PRKCE	AX-40988767, rs117329408, rs4968069, rs17034641, rs75701130	5.38
**Response to estrogen**	TPH2, F7, GHRL	rs3093253, rs41317118, rs117394235	**4.52**
**Positive regulation of growth hormone secretion**	KISS1, GHRL	rs11240694, rs117394235	**4.26**
Platelet activation	ITPR1, PRKCE, DGKD, PDPK1	rs17034641, rs2307068, rs75701130, rs3749383, AX-40988767, rs882428, rs76318740	4.09
Positive regulation of cell adhesion	MIP, VEGFA, APBB1IP	AX-38792903, rs3025039, rs3025022, AX-30823381	4.02

### Interpretation of the high performance of the preterm LBW models to clarify the influential genetic factors for prediction

Based on the SNP array data, the first and second highest performance interpretable models showed F1-scores of 0.89 and 0.77 for models using maternal SNP array data (Supplemental Table 7). Gene enrichment analysis showed a high importance in gene functions related to the toll-like receptor signaling pathway, and to the antimicrobial response in the preterm LBW model (Table 3). As same as the term LBW model, the GO terms from the first to the tenth importance score are shown in Table 3. All results of gene enrichment analysis for the preterm LBW model are shown in Supplemental Table 9. These results showed that the variants related to fetal growth were not influential in this model, as they were in the term LBW model, but that both intrinsic regulation of inflammation and response to microbes were influential in the preterm LBW model. Fetal inflammation triggered by bacterial invasion is known to be inversely related to gestational age at delivery [24], and these variants should shorten the gestational age that leads to LBW in the preterm birth group. Compared with the term LBW model, several GO terms showed high summary weights. This result may reflect higher performance of preterm LBW models based on genetic factors than that of term LBW models. We confirmed that all GO terms in Table 3 were significant based on only maternal variants, excepting paternal variants.

**Table 3:** Genes and their GO terms of the responsible variants for the preterm LBW prediction

**GO terms**	**Genes**	**Variants**	**The proportion of Importance score (%)**
**Negative regulation of toll-like receptor signaling pathway**	IRF4^a^, NLRP6, PDPK1	AX-30019939, rs76318740, rs2666970^a^	**11.19**
**Antimicrobial humoral response**	AZU1, DEFA4	AX-36710985, rs12460114	**5.01**
Cellular extravasation	AZU1, ITGA1	rs2456207, rs12460114, AX-15065431	4.69
Extracellular matrix organization	ITGA9, CRISPLD2, MADCAM1, NID1, TNR, ITGA1, SMOC2	rs2227163, AX-12439132, rs2171027, rs2456207, rs12599043, AX-30715297, rs6796546, rs3745925, AX-41915613, AX-15065431	3.73
Positive regulation of protein secretion	APBB1, EXPH5, PPID	rs8396, AX-11523840, rs3741048, AX-39132915	3.47
Cellular response to drug	RANBP1, RECQL5, KCNQ1, CAD	AX-33756771, rs79886149, rs3864884, rs72631384	2.69
Protein complex assembly	TCEB2, SLC9A3R2, HES1, PPID, IL2RB	rs2368048, rs8396, rs229489, rs4036, AX-40011429	2.67
Cellular response to insulin stimulus	CAPN10, INHBB, USF1, LPIN1, PDPK1	AX-13593741, rs76318740, rs117098832, AX-33691969, AX-39117777	0.10
Late viral transcription	USF1,MON1B	AX-39117777,AX-12559658	0.09
Chondroitin sulfate proteoglycan biosynthetic process	B3GAT1,ACPL2,CYTL1	AX-14133852,AX-41486923,rs6439996,rs75219147	0.09

Note that the “^a^” indicate the genes and variants of paternal SNP array data for attention because we observed the overfitting in the prediction models using these variants (Supplemental Table 5)

### The performance and interpretation of term and preterm models based on fetal ultrasonography data

The F1-score of the prediction model based on fetal ultrasonography data was 0.11 and 0.25 lower than that based on health assessment data for the term birth group and based on SNP array data for the preterm birth groups, respectively. These results illustrate the impossibility of the large-scale collection of high-quality fetal ultrasound data that can predict LBW due to problems in data production such as differences in ultrasonography equipment among hospitals. The interpretation of models using fetal ultrasonography data is described in the “The interpretation of the term LBW models using fetal ultrasonography data” section and “The interpretation of the preterm LBW model using health assessment and fetal ultrasonography data" section of the Supplemental Document.

## Discussion

This study challenged the development of supervised AI models for early prediction of LBW using 21,581 mothers, their neonates and partners data from BirThree cohort study. Our first salient achievement is that precise LBW prediction based on stratification of subjects into term and preterm birth groups. The AI models based on both comprehensive lifestyle data in the early stage of pregnancy and maternal SNP array data showed the highest predictive performance in term and preterm birth groups, respectively (AUC: 0.96 and 0.95). Our prediction model could inform risk of LBW in the early stage of pregnancy and is expected to control LBW risk through early intervention and/or improvements of lifestyles of pregnancies. Second crucial result is that the interpretation of models yielded influential features for both term and preterm birth groups. The influential features were radically different between the two groups: variables related to eating habits and variants related to fetal growth in the term birth group and variants related to inflammatory response and antimicrobial humoral response in the preterm birth group. Our findings contribute to a deeper understanding of the mechanism of LBW and are expected to be candidate biomarkers and new therapeutic targets of LBW.

### The term LBW model helps risk control of LBW through early intervention and improvements of lifestyles

In this study, we developed early prediction models of LBW in the term and preterm birth groups, named the term LBW model and preterm LBW model, respectively. The performance of the term LBW model reached AUC of 0.96 based on questionnaire data completed in the early stage of pregnancy. Because the mean gestational age at completion was 20.64 gestational weeks (6.54 SD), this result showed the possibility of early information of LBW risk to risk control through early intervention and improvements of lifestyles of pregnancies in the term birth group (Table 1). The term LBW model also showed a moderate performance based on fetal ultrasonography and SNP array data, with F1-scores up to 0.84 for both. The fetal ultrasonography data used in our study consisted of training data through 25 weeks of gestation and predicted data through 40 weeks of gestation by the LSTM model, showing the possibility of the prediction of LBW at 25 weeks of gestation using fetal ultrasonography data measured in usual care. For high-risk pregnancies identified by risk estimation based on lifestyles, a detailed assessment of fetal ultrasonography leads to more effective early intervention. There is still a possibility of improving the prediction performance by combining risk assessment based on both fetal ultrasonography data and environmental factors, and this investigation will be performed in the future study.

### The preterm LBW model showed the potential for early prediction of LBW based on genetic backgrounds

The preterm LBW model showed high performance with only the SNP array data, which reached AUC of 0.95. This result showed the possibility of the early estimation of the risk of LBW before pregnancy in the preterm birth group. While assessment of the LBW risk using genetic factors based on our established model is still not practical in clinical settings because our study is not clinical level but research level. To realize the early prediction of LBW in clinical settings, more research including clinical trials are required. Furthermore, a new era in which all patients have their own individual genomic information is forecasted to arrive in the near future [25]. In this new era, there is a possibility of the routine screening of LBW in the preterm birth group based on genomic information in a clinical setting.

### The eating habits, disease history and genetic factors related to fetal growth are influential for predicting LBW in the term birth group

In the term LBW models using questionnaire data, we identified eating habits as the most influential health assessment category because of the dominant high importance (29.17–84.16%). This result is reasonable because poor nutritional status and specific dietary patterns such as preconceptional high fat and sugar diet are known risk factors for LBW [26, 27]. Among the relatively high-importance categories other than eating habits, disease history including endometriosis, polycystic ovary syndrome and ovarian tumor/ovarian cysts showed high importance. Interestingly, a disease history of endometriosis has already been reported as a risk factor for LBW [28]. The subjects who had an ovarian tumor/ovarian cyst history were cancer survivors, and an increased LBW risk among cancer survivors has been reported in a previous study [29]. As for genetic factors, gene functions related to the secretion and regulation of hormones that are known to contribute to fetal growth [30–32], such as “positive regulation of insulin secretion”, “response to estrogen” and “positive regulation of growth hormone secretion”, showed high importance. These influential features contribute to identifying new therapeutic targets of LBW in the term birth group. Previous studies have used only environmental factors, whereas our study is the first to use both genetic and environmental factors, and develop precise early prediction models of LBW in the term group. Therefore, our study warrants further studies to clarify the effects of the interaction of genetic and environmental factors, such as the interactions between these variants and maternal low-calorie diets. The investigation of their interactions is a future grand challenge.

### Genetic factors related to inflammatory activity and antimicrobial response are influential for predicting LBW in the preterm birth group

In the preterm LBW models, as for genetic factors, the gene functions in the regulation of Toll-like receptor signaling pathway, showed high importance in the preterm LBW model. The Toll-like receptor signaling pathway is a gatekeeper of inflammatory activity, and relations for preterm birth have been reported [33]. Gene functions related to the antimicrobial humoral response also showed high importance in the preterm LBW model. Infections are known as one of the major causes of preterm birth [34], and variants related to the antimicrobial humoral response may be associated with LBW in the preterm birth group by affecting gestational age at birth. Shorter gestational age is known as having association with lower brain volume, cognitive and educational performance in early adolescence, and the lower brain volume still remains at 11 years old [35]. Thus, these variants will be new research targets to clarify the detailed molecular mechanisms of LBW and new therapeutic targets to prevent lower gestational age in the preterm birth group.

### Future work

We accomplished to establish the prediction models for single modal data for both preterm and term birth groups. For the preterm birth group, the models based on SNP array data were well-performed. The interpretation of models reveals the important gene functions for LBW including the toll-like receptor signaling pathway and the antimicrobial response. As for the term birth group, we observed high-performance in the models based on health assessment data. The interpretation reveals that eating habits showed dominant high importance for LBW.

Based on our crucial findings, we are planning to focus on establishing the multimodal prediction models based on genetic and environmental factors to handle the gene-environmental interactions.

### Limitations

This study has several limitations. Firstly, we were not able to evaluate the performance of models based on paternal SNP array data in the preterm birth group because of the overfitting. This overfitting is caused by paternal small sample size in the preterm birth group. Secondly, we did not consider genetic and environmental interactions because of extreme complexity and difficulty to develop adequate AI models. Thirdly, this study cannot perform external validation using other cohorts. This limitation is because of a lack of comparable large-scale cohorts that recruit both pregnancies and their partner and collect both comprehensive environmental and genetic factors.

## Conclusion

To our best knowledge, this is the first challenge to develop precise and generalized early prediction models of LBW based on both comprehensive environmental and genetic factors collected from over 23,000 pregnancies. Because of the differences in the mechanism of LBW in preterm and term birth groups, we wish to develop both preterm LBW and term LBW models based on the stratification of subjects by gestational age. The performance of the early prediction models reached AUC 0.95 and 0.96 for preterm LBW and term LBW models, respectively, and F1-scores of 0.90 for the preterm LBW and 0.95 for term LBW models based on genetic and environmental factors, respectively. Because of the accuracy and generalisability, our prediction model is expected to assess risk of LBW in the early stage of pregnancy and control LBW risk through improvements of lifestyles of pregnancies in the term birth group. Our prediction model is also expected to contribute precise prediction of LBW based on genetic factors in the preterm birth group if pregnancies and their partners know their own personal genetic data in the upcoming future.

Interpretation of the preterm LBW models identifies the influential variants for prediction. The gene functions of these variants are regulations of inflammation and antimicrobial humoral response, which may be involved in LBW through shortening gestational age due to fetal inflammation triggered by bacterial invasion. These variants are expected to be a new research target to clarify the detailed mechanisms of LBW in the preterm birth group. While in the term LBW model, variants about fetal growth among genetic factors and eating habits that can be controlled by patients were influential for prediction among comprehensive genetic and environmental factors. These influential features are expected to be major targets for understanding genetic and environmental contribution for LBW to address serious burdens on newborn’s health throughout life triggered by LBW.

### Supplementary Information

**Additional file 1.** Supplemental Document. **Supplemental Figure 1.** Scheme of the all analyses performed in our study. **Supplemental Figure 2.** The proportion of the importance score of the term LBW models. **Supplemental Figure 3.** The proportion of the importance score of the preterm LBW models. **Supplemental Figure 4.** The proportion of the importance score of the preterm LBW models. **Supplemental Figure 5.** F1-scores with randomly selected variables. **Supplemental Table 1.** The list of variables of the health assesment data. **Supplemental Table 2.** The datasets for both early and full-term prediction models. **Supplemental Table 3.** List of health assessment variables included in the models. **Supplemental Table 4.** The F1-scores for both early- and full-term prediction models. **Supplemental Table 5.** Details of the performance of early prediction models based on SNP array data. **Supplemental Table 6.** The feature importances of the early prediction models based on health assesment data. **Supplemental Table 7.** The feature importances of the early prediction models based on SNP array data. **Supplemental Table 8.** The gene enrichment analysis for the term LBW model. **Supplemental Table 9.** The gene enrichment analysis for the preterm LBW model. **Supplemental Table 10.** The proportion of the datasets. **Supplemental Table 11.** Number of selected features by feature selection. **Supplemental Table 12.** The feature importances of the early prediction models based on fetal ultrasonography data. **Supplemental Table 13.** The performance of the bagging models. **Supplemental Table 14.** The performance of the positive/negative controls. **Supplemental Table 15.** The previously reported locus among variants in the early prediction models. **Supplemental Table 16.** Summary of the population distribution of datasets.

## Data Availability

The data that support the findings of this study are available from Tohoku Medical Megabank project but restrictions apply to the availability of these data, which were used under license for the current study, and so are not publicly available. Data are however available from the authors upon reasonable request and with permission of the Tohoku Medical Megabank project (contact: dist@megabank.tohoku.ac.jp).

## Abbreviations

BirThree Cohort Study: The Birth and Three-Generation Cohort Study
TMM Project: Tohoku Medical Megabank Project
LBW: Low birth weight
SGA: Small for gestational age
AI: Artificial intelligence
JPAv2: Japonica array v2
JPA NEO: Japonica array NEO
EFBW: Estimated fetal body weight
QC: Quality control
HSIC-LASSO: Hilbert–Schmidt independence criterion least absolute shrinkage and selection operator
RFE: Recursive feature elimination
LR: Logistic regressions
RF: Random forest
SVM: Support vector machine
DNN: Deep neural network
XGBoost: Extreme gradient boosting
AUC: Area under the curve
FGR: Fetal growth restriction

## References

[1] Cutland CL, Lackritz EM, Mallett-Moore T, Bardají A (2017). Low birth weight: case definition & guidelines for data collection, analysis, and presentation of maternal immunization safety data. Vaccine.

[2] de ValeroBernabé J, Soriano T, Albaladejo R, Juarranz M, Calle ME, Martínez D (2004). Risk factors for low birth weight: a review. Eur J Obstet Gynecol Reprod Biol.

[3] Warrington NM, Beaumont RN, Horikoshi M, Day FR, Helgeland Ø, Laurin C (2019). Maternal and fetal genetic effects on birth weight and their relevance to cardio-metabolic risk factors. Nat Genet.

[4] Horikoshi M, Beaumont RN, Day FR, Warrington NM, Kooijman MN, Fernandez-Tajes J (2016). Genome-wide associations for birth weight and correlations with adult disease. Nature.

[5] Linsell L, Malouf R, Morris J, Kurinczuk JJ, Marlow N (2015). Prognostic factors for poor cognitive development in children born very preterm or with very low birth weight: a systematic review. JAMA Pediatr.

[6] Karimi M, Fallah R, Dehghanpoor A, Mirzaei M (2011). Developmental status of 5-year-old moderate low birth weight children. Brain Dev.

[7] Mu M, Wang S-F, Sheng J, Zhao Y, Li H-Z, Hu C-L (2012). Birth weight and subsequent blood pressure: a meta-analysis. Arch Cardiovasc Dis.

[8] Abel KM, Wicks S, Susser ES, Dalman C, Pedersen MG, Mortensen PB (2010). Birth weight, schizophrenia, and adult mental disorder: is risk confined to the smallest babies?. Arch Gen Psychiatry.

[9] Collins FS, Varmus H (2015). A new initiative on precision medicine. N Engl J Med.

[10] Liao LD, Ferrara A, Greenberg MB, Ngo AL, Feng J, Zhang Z (2022). Development and validation of prediction models for gestational diabetes treatment modality using supervised machine learning: a population-based cohort study. BMC Med.

[11] Johnson KB, Wei W-Q, Weeraratne D, Frisse ME, Misulis K, Rhee K (2021). Precision medicine, AI, and the future of personalized health care. Clin Transl Sci.

[12] Ahmadi P, Alavimajd H, Khodakarim S, Tapak L (2017). Prediction of low birth weight using random forest: a comparison with logistic regression. Arch Adv.

[13] Saw SN, Biswas A, Mattar CNZ, Lee HK, Yap CH (2021). Machine learning improves early prediction of small-for-gestational-age births and reveals nuchal fold thickness as unexpected predictor. Prenat Diagn.

[14] Kuriyama S, Metoki H, Kikuya M, Obara T, Ishikuro M, Yamanaka C (2020). Cohort profile: Tohoku medical megabank project birth and three-generation cohort study (TMM BirThree cohort study): rationale, progress and perspective. Int J Epidemiol.

[15] Ogishima S, Nagaie S, Mizuno S, Ishiwata R, Iida K, Shimokawa K (2021). dbTMM: an integrated database of large-scale cohort, genome and clinical data for the Tohoku medical megabank project. Human Genome Variation.

[16] Hunter DJ (2005). Gene–environment interactions in human diseases. Nat Rev Genet.

[17] Kawai Y, Mimori T, Kojima K, Nariai N, Danjoh I, Saito R (2015). Japonica array: improved genotype imputation by designing a population-specific SNP array with 1070 Japanese individuals. J Hum Genet.

[18] Sakurai-Yageta M, Kumada K, Gocho C, Makino S, Uruno A, Tadaka S (2021). Japonica Array NEO with increased genome-wide coverage and abundant disease risk SNPs. J Biochem.

[19] Sugawara J, Ishikuro M, Obara T, Onuma T, Murakami K, Kikuya M (2020). Maternal baseline characteristics and perinatal outcomes: the Tohoku medical megabank project birth and three-generation cohort study. J Epidemiol.

[20] van Buuren S, Groothuis-Oudshoorn K (2011). mice: multivariate imputation by chained equations in R. J Stat Software Art.

[21] Yeo I, Johnson RA (2000). A new family of power transformations to improve normality or symmetry. Biometrika.

[22] Yamada M, Jitkrittum W, Sigal L, Xing EP, Sugiyama M (2014). High-dimensional feature selection by feature-wise kernelized Lasso. Neural Comput.

[23] Chen T, Guestrin C (2016). XGBoost: a scalable tree boosting system. In: proceedings of the 22nd ACM SIGKDD international conference on knowledge discovery and data mining.

[24] Kemp MW (2014). Preterm birth, intrauterine infection, and fetal inflammation. Front Immunol.

[25] Singh S (2018). The hundred-dollar genome: a health care cart before the genomic horse. CMAJ.

[26] Ramakrishnan U (2004). Nutrition and low birth weight: from research to practice. Am J Clin Nutr.

[27] Grieger JA, Grzeskowiak LE, Clifton VL (2014). Preconception dietary patterns in human pregnancies are associated with preterm delivery. J Nutr.

[28] Yi KW, Cho GJ, Park K, Han SW, Shin J-H, Kim T (2020). Endometriosis is associated with adverse pregnancy outcomes: a national population-based study. Reprod Sci.

[29] Anderson C, Engel SM, Mersereau JE, Black KZ, Wood WA, Anders CK (2017). Birth outcomes among adolescent and young adult cancer survivors. JAMA Oncol.

[30] Gatford KL, Simmons RA (2013). Prenatal programming of insulin secretion in intrauterine growth restriction. Clin Obstet Gynecol.

[31] Albrecht ED, Pepe GJ (2010). Estrogen regulation of placental angiogenesis and fetal ovarian development during primate pregnancy. Int J Dev Biol.

[32] Oberbauer AM (2015). Developmental programming: the role of growth hormone. J Anim Sci Biotechnol.

[33] Robertson SA, Hutchinson MR, Rice KC, Chin P-Y, Moldenhauer LM, Stark MJ (2020). Targeting Toll-like receptor-4 to tackle preterm birth and fetal inflammatory injury. Clin Transl Immunol.

[34] Wadhwa PD, Culhane JF, Rauh V, Barve SS, Hogan V, Sandman CA (2001). Stress, infection and preterm birth: a biobehavioural perspective. Paediatr Perinat Epidemiol.

[35] Ma Q, Wang H, Rolls ET, Xiang S, Li J, Li Y (2022). Lower gestational age is associated with lower cortical volume and cognitive and educational performance in adolescence. BMC Med.

