# Supplementary material for: Establishment of the early prediction models of low-birth-weight reveals influential genetic and environmental factors: a prospective cohort study
Source: BMC Pregnancy Childbirth. 2023 Aug 31;23:628. doi: 10.1186/s12884-023-05919-5 (PMC10472725; doi:10.1186/s12884-023-05919-5)
Supplement: Supplementary file 1 — Additional file 1. Supplemental Document. Supplemental Figure 1. Scheme of the all analyses performed in our study. Supplemental Figure 2. The proportion of the importance score of the term LBW models. Supplemental Figure 3. The proportion of the importance score of the preterm LBW models. Supplemental Figure 4. The proportion of the importance score of the preterm LBW models. Supplemental Figure 5. F1-scores with randomly selected variables. Supplemental Table 1. The list of variables of the health assesment data. Supplemental Table 2. The datasets for both early and full-term prediction models. Supplemental Table 3. List of health assessment variables included in the models. Supplemental Table 4. The F1-scores for both early- and full-term prediction models. Supplemental Table 5. Details of the performance of early prediction models based on SNP array data. Supplemental Table 6. The feature importances of the early prediction models based on health assesment data. Supplemental Table 7. The feature importances of the early prediction models based on SNP array data. Supplemental Table 8. The gene enrichment analysis for the term LBW model. Supplemental Table 9. The gene enrichment analysis for the preterm LBW model. Supplemental Table 10. The proportion of the datasets. Supplemental Table 11. Number of selected features by feature selection. Supplemental Table 12. The feature importances of the early prediction models based on fetal ultrasonography data. Supplemental Table 13. The performance of the bagging models. Supplemental Table 14. The performance of the positive/negative controls. Supplemental Table 15. The previously reported locus among variants in the early prediction models. Supplemental Table 16. Summary of the population distribution of datasets. [file 12884_2023_5919_MOESM1_ESM.zip › Additional file 1/Supplemental Document.pdf]

Supplemental Methods

Evaluation of sampling bias, possibility of improving the performance and extreme overfitting/underfitting

The performance of the early prediction model was compared to that of the random sampling plus bagging model to evaluate the possibility of sampling bias in undersampling, and controls to evaluate the possibility of extreme overfitting/underfitting. The full-term prediction model was developed from all of the data collected in the baseline period other than those for the early stage of pregnancy to evaluate the possibility of improving the performance of the early prediction model by adding data. All 14 datasets for the full-term prediction model are shown in Supplemental Table 2.

Building datasets of health assessment datasets

Before building datasets, we removed fetal ultrasonography data from prenatal checkup datasets. From the prenatal checkup dataset, we created three sub datasets with window sizes of 5 weeks and 13 weeks called prenatal checkup datasets 1 and 2, respectively. For the development of the early prediction models, laboratory test data collected in the early stage of pregnancy and questionnaires completed in the early stage of pregnancy were used. For the full-term prediction model, we used all of the available health assessment data, including laboratory tests, questionnaires and medical records. The three sub datasets of prenatal checkup data with different window sizes were combined with all of the remaining data to create three concatenated datasets, which were named concatenated datasets 1 and 2. In the creation of the prenatal checkup datasets, the average value was taken for multiple measurements in the same gestational week.

Imputation of missing values

We applied imputation of missing values using multiple imputation by chained equations (MICE) [1]. Before imputation with the MICE algorithm, we removed subjects with a high missing rate (greater than 70%) and variables with missing rates greater than 50% from the datasets.

Conversion of distribution

We applied Box-Cox conversion [2], scaling and Box-Cox conversion plus scaling for conversion of distribution, and we adapted the most high-performance data in prediction within three types of conversion of distribution as the final datasets.

Items of fetal ultrasonography data used

We used the following 13 items of the fetal ultrasonography data from prenatal checkups: gestational sac (GS), cervical length (CL), crown-rump length (CRL), biparietal diameter (BPD), abdominal circumstance (AC), transverse trunk diameter (TTD), anteroposterior trunk diameter (APTD), fetal trunk area (FTA), fetal femoral length (FFL), estimated fetal body weight (EFBW), amniotic fluid index (AFI), maximum vertical pocket (MVP), and amniotic fluid pocket (AFP).

Quality control of the fetal ultrasonography data

We performed the following QC steps for the fetal ultrasonography data: 1) remove CRL values before 18 weeks of pregnancy and GS data after 23 weeks of pregnancy, 2) correct obvious unit errors in micrometers and millimeters, 3) remove negative values, 4) calculate missing EFBW values using BPD, AC and FL values if applicable and 5)

remove outliers of mean±4 SD.

Spline interpolation of the fetal ultrasonography data

We performed spline interpolation of the fetal ultrasonography data by the Akima interpolation method [3] using the function of Scipy [4].

Multiple imputation of the fetal ultrasonography data

We performed multiple imputation of missing values to impute values of the segment with no data that cannot be interpolated by spline interpolation using MICE [1]. Before multiple imputation, we removed subjects with a high missing rate of more than 70% and items with a high missing rate of more than 60%. We performed further spline interpolation of missing values that were not imputed by multicollinearity to build complete data.

Prediction of fetal ultrasonography data at the late stage of pregnancy using data in the early stage of pregnancy

To build fetal ultrasonography data for the early prediction models, we predicted values for the late stage of pregnancy from those of the early stage of pregnancy. We built the following two datasets with different periods of pregnancy as training data using the long short-term memory (LSTM) [5] network: 1) training data from 10 to 31 weeks of gestation and predicted data for the remaining gestational weeks (Fetal ultrasonography data (training data : 10-31 weeks of gestation)), and 2) training data from 10 to 25 weeks of gestation and predicted data for the remaining gestational weeks (Fetal ultrasonography data (training data : 10-25 weeks of gestation)). The developed LSTM

model was a three-layered model with 200 epochs for training. We input scaled data into the LSTM model and obtained final values by inverse transform of the predicted values. We used Chainer [6, 7] version 5.0.0 for the development of the LSTM model.

Building SNP array data

We used maternal and paternal SNP array data for early prediction models, and neonatal SNP array data for full-term prediction models. For the full-term prediction model, we also merged maternal, paternal, and neonatal SNP array data to build the merged data.

Sample-based and probe-based quality control of SNP array data

QC for the SNP array data was performed as follows: 1) remove variants with under 0.01 of minor allele frequency (MAF), 2) remove subjects with over 0.5 of inbreeding coefficient (pi-hat), 3) remove subjects with inconsistent estimated sex from the SNP array data and registered sex in the cohort, 4) remove subjects who answered that their birthplace was other than Japan in self-reported questionnaires, 5) remove subjects with under 0.99 of call rate, 6) remove variants with under 0.99 of call rate, and 7) remove deviated variants from Hardy-Weinberg equilibrium (HWE). We used $P < 1 \times 10^{-6}$ as the threshold of the HWE test. PLINK 1.9beta was used for QC of the SNP array data.

Dimension reduction of the SNP array data

The dimension reduction of the SNP array data was performed as follows: 1) pruning of variants for linkage disequilibrium (LD) and 2) filtering of variants using *p*-values of genome-wide association analysis. We performed LD pruning with $R^2 < 0.1$ as the threshold. We selected variants with $p < 0.05$ in the allelic test with Fisher's exact test in

the LBW analysis for all the prediction models.

Feature selection

In this study, we adapted the Hilbert-Schmidt independence criterion least absolute shrinkage and selection operator (HSIC-LASSO) [8] as well as recursive feature elimination (RFE) [9] using the RFE-CV function of scikit-learn [10] for feature selection. HSIC-LASSO was used to obtain the set of dependent features with labels and low redundancy in a nonlinear space. RFE obtains a set of features with the highest prediction performance by weighting the features with an external estimator and repeatedly removing the variables with the smallest weights.

Sampling for imbalanced learning

In this study, we compared the performances between the prediction models with undersampling using the NearMiss-1 algorithm [11] and bootstrap aggregating (bagging), along with random sampling to evaluate the effect of sampling bias. For the development of the random sampling model, we used imblearn in Python 3. For the development of the bagging model, XGBoost [12] was utilized as the learning model.

Development of the machine learning models

To obtain a model with optimal performance, we built five machine learning models for all the datasets as follows: 1) logistic regression (LR), 2) random forest (RF) [13], 3) support vector machine (SVM) [14], 4) deep neural network (DNN) and 5) XGBoost [12]. Scikit-learn [10] version 0.20 was used to develop the LR, RF and SVM models. Chainer [6, 7] version 5.0.0 was used to build the DNN model. The radial basis function (RBF)

kernel was adapted for the SVM classifier. The parameters of the LR, RF, SVM and XGBoost models were optimized by maximizing the F1-score with grid search. The optimized parameters of the DNN were obtained by maximizing the F1-score with sequential model-based global optimization (SMBO) [15] using the hyperopt [16] library. To avoid overfitting and to fix outliers in the datasets, we adopted internal-external cross-validation [17]. In this technique, we used one-tenth of the dataset as validation data, and performed 10-fold cross-validation using remaining data. We repeated this step 10 times and took the average of F1-scores as the performance of the established model.

Interpretation of the developed prediction models

We obtained feature importance from the first and second high-performance models among the interpretable LR, RF and XGBoost models to interpolate the developed early prediction models. We used both the normalized absolute regression coefficients of the LR model by dividing by the sum of regression coefficients of all features and the Gini coefficient of RF and XGBoost as feature importance. From the importance, we obtained categories of the features and their proportion of importance scores among all features. We performed further analysis for the first and second highest performance prediction model using maternal and paternal SNP array data to perform precise interpretation as follows: 1) genomic mapping to obtain the gene type (e.g., protein coding) and nearest gene names, 2) functional enrichment analysis using variants without in introns regions, in no gene regions and in noncoding intron regions, 3) summary weight calculation for the enriched functions, 4) evaluation of the differences in the enriched gene functions between the preterm LBW model and term LBW model and 5) evaluation of the importance of loci that have been previously reported to be associated with LBW and

birthweight among the variants input into the model. We performed genomic mapping using the SNP nexus [18] with Ensemble. Gene enrichment analysis was performed through DAVID [19] using the nearest gene names of the variants. We calculated the summary weights for the enriched functions as follows: 1) obtained the percentage of importance of variants among all variants input into the model, 2) obtained sum of values in step 1 for each enriched function and 3) calculated the summary weights by dividing the values obtained in step 2 by the number of variants for each enriched gene function. From the calculated summary weights, we obtained the importance scores for gene function about GO bioprocesses by normalization so that the sum of the importance of these gene functions is 1. We defined the variants within 500 kilobases (kb) apart and in LD with $r^2 \geq 0.1$ as same loci.

Building the positive and negative controls

In this study, we created 100-1200 variables with high correlation coefficients of 0.60-0.95 with labels for positive controls. Negative controls were created by randomizing the labels of the datasets.

Supplemental Results

Number of features, subjects and mean of collected gestational weeks in each dataset

The number of subjects, features, LBW subjects and mean gestational weeks of data collected for each dataset in the early prediction model are shown in Table 1. The number of subjects, features, LBW neonates and their parents for all datasets are shown in Supplemental Table 10

Number of selected features by feature selection

The number of features selected by feature selection is provided in Supplemental Table 11. The feature selection of the fetal ultrasonography data by HSIC LASSO was not convergent, and all items were input into machine learning models without feature selection.

The interpretation of the term LBW models using fetal ultrasonography data

The first- and second-best performance models among interpretable LR, RF and XGBoost models, the F1-scores were 0.79 and 0.80 in both XGBoost models. The interpretation of those models showed that EFBW and AC showed high importance, which were 32.44- 45.73% and 24.46-31.61%, respectively (Supplemental Figure 2). The high importance of AC is reasonable because of the linear relation between AC and EFBW [20]. The feature names, feature importance and category of features for all the features based on the fetal ultrasonography data are shown in Supplemental Table 12.

The interpretation of the preterm LBW model using health assessment and fetal ultrasonography data

Among the health assessment data, the F1-scores of the preterm LBW models using questionnaire data were lower (0.52-0.62) than models using laboratory tests data (0.59-0.71). The performance of the first- and second-best performance interpretable models based on health assessment data were 0.63 and 0.64, and those models were based on laboratory tests data. The interpretation of those models showed that immunological tests (47.28-90.74%) showed dominant high importance (Supplemental Figure 3).

Except for features about immunological tests, features about urinalysis (9.02% in RF model) or biochemical test (44.41 in LR model) showed relatively high importance in the second-best performance model. The high importance of features of immunological tests was reasonable because an association between impairment of the regulation of proinflammatory responses and LBW has been reported in a previous study [21]. Based on fetal ultrasonography data, F1-scores of the first- and second-best interpretable models were both 0.69. The interpretation of those models showed that EFBW and AC showed high importance in the RF model as same as term LBW models, which were 39.27 and 21.98%, respectively. Differ from term LBW models, BPD and FFL showed high importance in the LR model, which were 45.21 and 34.06% (Supplemental Figure 4). The feature names, feature importance and category of features for all the features based on the fetal ultrasonography data are shown in Supplemental Table 12.

Performance of the bagging models

The performance of the bagging models in the early prediction models reached the F1-scores reached 0.55, 0.68 and 0.65 based on the health assessment data, SNP array data and fetal ultrasonography data, respectively, in the preterm LBW model. In the term LBW model, the performance reached F1-scores of 0.78, 0.92 and 0.74 based on the health assessment data, SNP array data and fetal ultrasonography data, respectively. The performances of all the bagging models are shown in Supplemental Table 13

Performance of positive control and negative control

The performance of the positive control was 1.0 for all the models. The F1-scores of the negative controls reached 0.64 and 0.57 for the preterm LBW and term LBW models,

respectively. All performances of the positive controls and negative controls are shown in Supplemental Table 14.

Performance of the full-term prediction models

The performance of the full-term prediction models reached F1-scores of 0.92 and 0.98 for the preterm LBW and term LBW models, respectively. There were few differences in performance compared to the early prediction models (-0.03 and +0.03 of F1-scores for preterm LBW models and term LBW model, respectively). The performance of all the full-term prediction models is shown in Supplemental Table 4.

Supplemental Discussion

The prediction of fetal ultrasonography data in late stage of pregnancy using early stage of pregnancy data

The fetal ultrasonography data used in our study consisted of training data through 25 weeks of gestation and predicted data through 40 weeks of gestation by the LSTM model. In the first and second best-performing interpretable models among the five machine learning models, the EFBW values predicted by LSTM accounted for 32.33–85.40% of the importance of all EFBW values. This result showed the capability of precisely predicting the fetal ultrasonography data of the late stage of pregnancy using data from the early stage of pregnancy.

The previously reported locus among variants in the early prediction models

The variants in the early prediction models included loci associated with LBW or birth weight reported in previous studies [22–24] (Supplemental Table 15). For the maternal

SNP array data, the preterm LBW model included 5 loci, and the term LBW model included 1 locus previously reported. The percentage of these variants among all variants in each model was 0.007-0.37%. For the paternal SNP array data, the preterm LBW model included 2 loci, and the term LBW model included 3 loci previously reported. The percentage of these variants among all the variants in each model was 0.06-1.96%. Among the previously reported loci in the maternal SNP array data, a locus coding LEKR1/CCNL1 showed relatively high importance, and the association with glucose-stimulated insulin release levels with this locus has been reported in a previous study [25]. A highly important locus in the paternal SNP array data coding PROX1 is known as a critical transcription factor in organ development during embryogenesis [26]. In addition to these loci, other highly important variants among the maternal and paternal SNP array data may have roles in various events, including fetal development.

Comparison of the performance of the early- and full-term prediction models

The prediction performance of the full-term prediction models reached F1-scores of 0.92 and 0.98 in the preterm LBW model and term LBW model, respectively. The improvements in the F1-scores in the full-term prediction model compared with the early prediction model was 0.03 and 0.08 in the term LBW models and the preterm LBW model, respectively. The lack of improvement in the preterm LBW model suggested that no features other than parental genomic features contributed to the prediction of LBW in the preterm birth group. Small improvement in the F1-scores of the term LBW model may be caused by inclusion of blood pressure, which is related to hypertension, which is known as a risk factor for LBW in the term birth group in the concatenated dataset. The small improvement in the F1-scores in the full-term prediction model showed that the

performance of the early prediction model cannot be significantly improved by adding data.

Comparison of the performance of the bagging models

The performance of the bagging models reached F1-scores of 0.65 and 0.92 in the preterm LBW model and term LBW model, respectively. The performance was decreased by 0.25, and 0.03 in the preterm LBW model and term LBW model, respectively, compared with that of the early prediction model. The high performance of the bagging model in term LBW model may be caused by overfitting because the F1-scores increased as the number of randomly selected features increased based on the input data and maternal SNP array data (JPAv2) (Supplemental figure 5). This result showed that undersampling plus feature selection improved the performance more than random sampling plus bagging.

References

1. van Buuren S, Groothuis-Oudshoorn K. mice: Multivariate Imputation by Chained Equations in R. Journal of Statistical Software, Articles. 2011;45:1–67.

2. Yeo I, Johnson RA. A new family of power transformations to improve normality or symmetry. Biometrika. 2000;87:954–9.

3. Akima H. A new method of interpolation and smooth curve fitting based on local procedures. J ACM. 1970.

4. Virtanen P, Gommers R, Oliphant TE, Haberland M, Reddy T, Cournapeau D, et al. SciPy 1.0: fundamental algorithms for scientific computing in Python. Nat Methods. 2020;17:261–72.

5. Hochreiter S, Schmidhuber J. Long Short-Term Memory. Neural Comput. 1997;9:1735–80.

6. Tokui S, Oono K, Hido S, Clayton J. Chainer: a next-generation open source framework for deep learning. In: Proceedings of workshop on machine learning systems (LearningSys) in the twenty-ninth annual conference on neural information processing systems (NIPS). learningsys.org; 2015. p. 1–6.

7. Akiba T, Fukuda K, Suzuki S. ChainerMN: Scalable Distributed Deep Learning Framework. arXiv [cs.DC]. 2017.

8. Yamada M, Jitkrittum W, Sigal L, Xing EP, Sugiyama M. High-dimensional feature selection by feature-wise kernelized Lasso. Neural Comput. 2014;26:185–207.

9. Guyon I, Weston J, Barnhill S, Vapnik V. Gene Selection for Cancer Classification using Support Vector Machines. Mach Learn. 2002;46:389–422.

10. Pedregosa F, Varoquaux G, Gramfort A. Scikit-learn: Machine learning in Python. of machine Learning …. 2011.

11. Mani I, Zhang I. kNN approach to unbalanced data distributions: a case study involving information extraction. In: Proceedings of workshop on learning from imbalanced datasets. ICML United States; 2003.

12. Chen T, Guestrin C. XGBoost: A Scalable Tree Boosting System. In: Proceedings of the 22nd ACM SIGKDD International Conference on Knowledge Discovery and Data Mining. New York, NY, USA: Association for Computing Machinery; 2016. p. 785–94.

13. Ho TK. Random decision forests. In: Proceedings of 3rd International Conference on Document Analysis and Recognition. ieeexplore.ieee.org; 1995. p. 278–82 vol.1.

14. Cortes C, Vapnik V. Support-vector networks. Mach Learn. 1995;20:273–97.

15. Bergstra J, Bardenet R, Bengio Y, Kégl B. Algorithms for Hyper-Parameter Optimization. In: Shawe-Taylor J, Zemel R, Bartlett P, Pereira F, Weinberger KQ, editors. Advances in Neural Information Processing Systems. Curran Associates, Inc.; 2011.

16. Bergstra J, Yamins D, Cox D. Making a Science of Model Search: Hyperparameter Optimization in Hundreds of Dimensions for Vision Architectures. In: Dasgupta S, McAllester D, editors. Proceedings of the 30th International Conference on Machine Learning. Atlanta, Georgia, USA: PMLR; 2013. p. 115–23.

17. Steyerberg EW, Harrell FE Jr. Prediction models need appropriate internal, internal-external, and external validation. J Clin Epidemiol. 2016;69:245–7.

18. Oscanoa J, Sivapalan L, Gadaleta E, Dayem Ullah AZ, Lemoine NR, Chelala C. SNPnexus: a web server for functional annotation of human genome sequence variation (2020 update). Nucleic Acids Res. 2020;48:W185–92.

19. Huang DW, Sherman BT, Lempicki RA. Systematic and integrative analysis of large gene lists using DAVID bioinformatics resources. Nat Protoc. 2009;4:44–57.

20. Smith GC, Smith MF, McNay MB, Fleming JE. The relation between fetal abdominal circumference and birthweight: findings in 3512 pregnancies. Br J Obstet Gynaecol. 1997;104:186–90.

21. Gyllenhammer LE, Entringer S, Buss C, Simhan HN, Grobman WA, Adam EK, et al. Prospective association of maternal immune pro-inflammatory responsivity and regulation in pregnancy with length of gestation. Am J Reprod Immunol. 2021;85:e13366.

22. Mu M, Wang S-F, Sheng J, Zhao Y, Li H-Z, Hu C-L, et al. Birth weight and subsequent blood pressure: a meta-analysis. Arch Cardiovasc Dis. 2012;105:99–113.

23. Juliusdottir T, Steinthorsdottir V, Stefansdottir L, Sveinbjornsson G, Ivarsdottir EV, Thorolfsdottir RB, et al. Distinction between the effects of parental and fetal genomes on fetal growth. Nat Genet. 2021;53:1135–42.

24. Freathy RM, Mook-Kanamori DO, Sovio U, Prokopenko I, Timpson NJ, Berry DJ, et al. Variants in ADCY5 and near CCNL1 are associated with fetal growth and birth weight. Nat Genet. 2010;42:430–5.

25. Andersson EA, Harder MN, Pilgaard K, Pisinger C, Stančáková A, Kuusisto J, et al. The birth weight lowering C-allele of rs900400 near LEKR1 and CCNL1 associates with elevated insulin release following an oral glucose challenge. PLoS One. 2011;6:e27096.

26. Elsir T, Smits A, Lindström MS, Nistér M. Transcription factor PROX1: its role in development and cancer. Cancer Metastasis Rev. 2012;31:793–805.
