# Supplementary material for: Establishment of the early prediction models of low-birth-weight reveals influential genetic and environmental factors: a prospective cohort study
Source: BMC Pregnancy Childbirth. 2023 Aug 31;23:628. doi: 10.1186/s12884-023-05919-5 (PMC10472725; doi:10.1186/s12884-023-05919-5)

Supplemental Figure 2 : The proportion of the importance score of the term LBW models

Fetal ultrasonography data

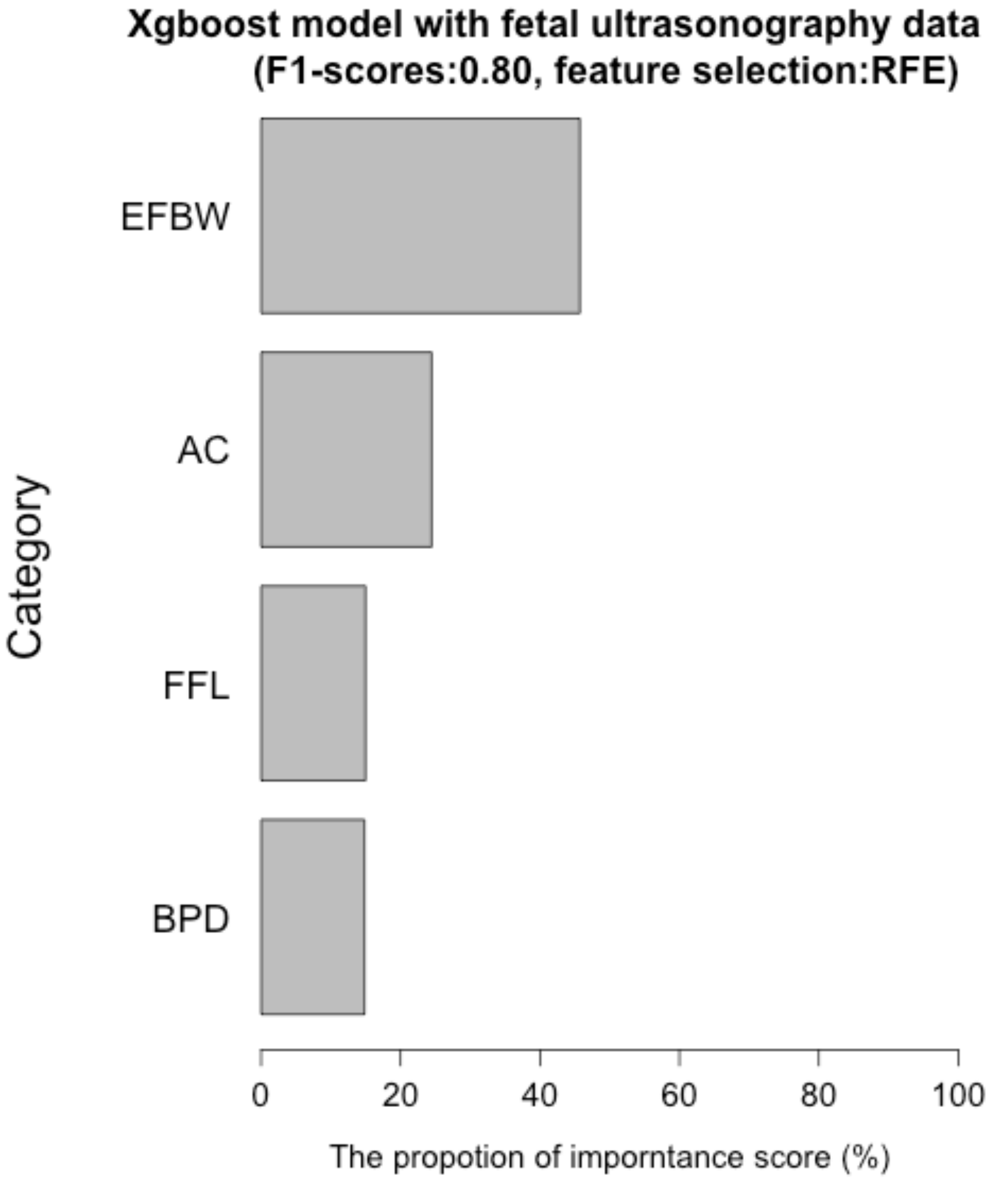

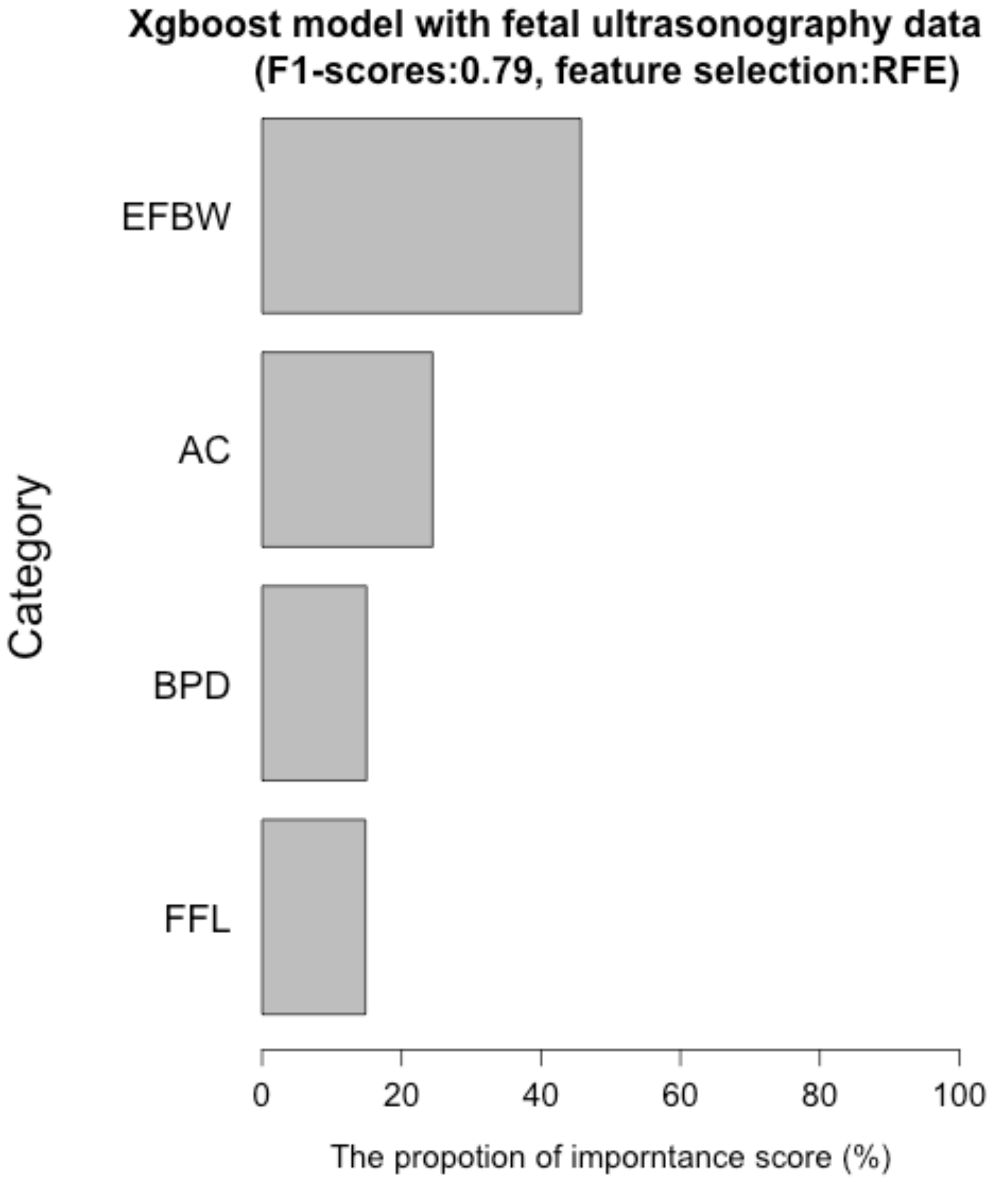

Supplement: Supplementary file 1 — Additional file 1. Supplemental Document. Supplemental Figure 1. Scheme of the all analyses performed in our study. Supplemental Figure 2. The proportion of the importance score of the term LBW models. Supplemental Figure 3. The proportion of the importance score of the preterm LBW models. Supplemental Figure 4. The proportion of the importance score of the preterm LBW models. Supplemental Figure 5. F1-scores with randomly selected variables. Supplemental Table 1. The list of variables of the health assesment data. Supplemental Table 2. The datasets for both early and full-term prediction models. Supplemental Table 3. List of health assessment variables included in the models. Supplemental Table 4. The F1-scores for both early- and full-term prediction models. Supplemental Table 5. Details of the performance of early prediction models based on SNP array data. Supplemental Table 6. The feature importances of the early prediction models based on health assesment data. Supplemental Table 7. The feature importances of the early prediction models based on SNP array data. Supplemental Table 8. The gene enrichment analysis for the term LBW model. Supplemental Table 9. The gene enrichment analysis for the preterm LBW model. Supplemental Table 10. The proportion of the datasets. Supplemental Table 11. Number of selected features by feature selection. Supplemental Table 12. The feature importances of the early prediction models based on fetal ultrasonography data. Supplemental Table 13. The performance of the bagging models. Supplemental Table 14. The performance of the positive/negative controls. Supplemental Table 15. The previously reported locus among variants in the early prediction models. Supplemental Table 16. Summary of the population distribution of datasets. [file 12884_2023_5919_MOESM1_ESM.zip › Additional file 1/Supplemental Figure 2.pdf]
